# Making life easier for people with low vision

**Published:** 2012

**Authors:** Clare Gilbert

**Affiliations:** Co-director, International Centre for, Eye Health, London School of Hygiene I and Tropical Medicine, Keppel Street, London WC1E 7HT, UK; Clinical Advisor, Sightsavers.

**Figure:**
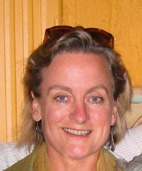
Clare Gilbert

There are many things you can suggest that will help people with low vision make the most of their vision, whether they are able to benefit from magnification devices or not.

If you are working at the community or at primary level, remember that these environmental modifications should never be a substitute for referral: always refer someone with low vison for an eye examination, refraction and low vision services wherever possible. If you are working at district/secondary or tertiary level, refer your patient for vision rehabilitation.

The suggestions given here are a good starting point, but some people may require further support and training in order to make the most of their vision.

A way of remembering environmental modifications is to think about:

- Making things **bigger** and **bolder**
- Using **colour** and **contrast**
- Improving **lighting**, using **lines**, and trying to **lift** what you want to look at.

## Bigger and bolder

Bringing things closer to our eyes makes them appear **bigger**. This mainly helps young people and children who have very good accommodation.

People (including children) who have had cataract surgery and those with presbyopia will need a near add (a plus lens) to bring things into focus if they bring them nearer.

**Figure 1.**
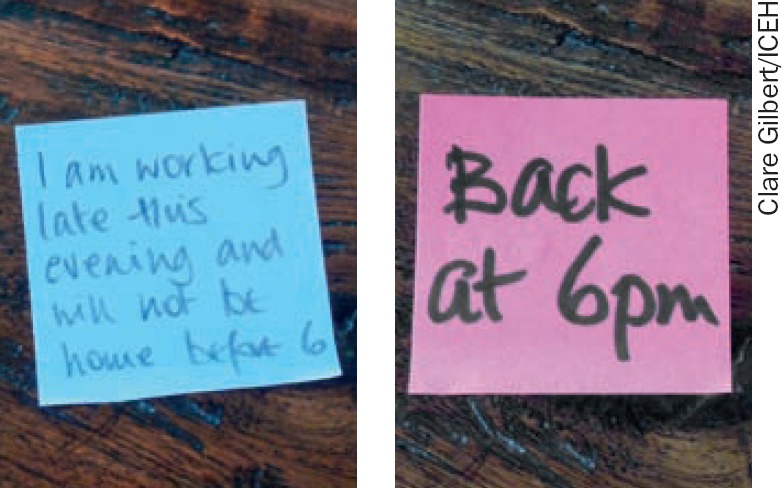
Bigger and bolder (right) Small writing (left) is not as easy to read as big, bold writing. Shorter is better.

Use charcoal or a felt pen to write **bolder** messages, and write with larger letters than usual (Figure 1). Keep it short and simple! Put it somewhere visible and write on a bright piece of paper if you want to attract the person's attention.

Enlarging photocopiers and computer screens are also ways that print and other images can be made bigger and hence easier for the person with low vision to see.

## Colour and contrast

**Figure 2.**
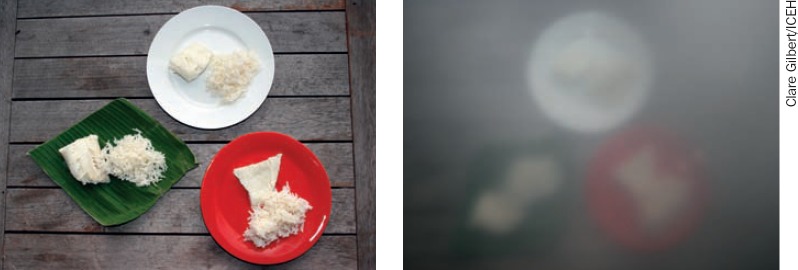
Using contrasting colours to improve visibility With normal vision, the rice is visible against all backgrounds. With low vision (right), the rice is much easier to see on the green banana leaf and red plate

**Colour** can be used in many ways to help someone in their home. For example:

- Use brightly coloured plates (Figure 2)
- Put red tape around light switches
- Use paint or red nail varnish to put spots of red to help the person line up the “off” buttons on the gas cooker
- Stand the person's shoes on a brightly coloured mat to distinguish them from other family members' shoes
- Mark the bottle of medication that is to be taken in the morning with a big yellow circle (to represent sunrise) and the evening bottle with a big black circle (to represent night).

**Figure 3.**
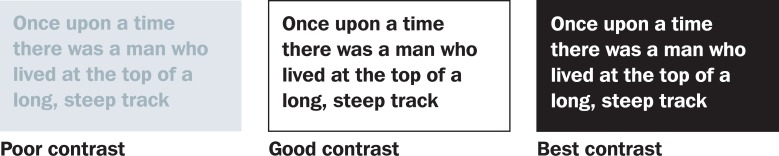
The effect of contrast on the legibility of text

**Contrast** makes things easier to see. For example, a black pen on white paper is easier to read than pencil. White writing on a black background gives the greatest contrast and hence is easier to read, but this can usually only be generated on a computer screen (Figure 3).

## Lighting, lines, and lift

**Lighting** is perhaps the best way to improve contrast, so if someone wants to read make sure the page is well lit. Ideally, the light should shine directly onto the page, but without producing glare. It should not shine in their eyes. Good lighting in darker areas of the home is important, particularly where the person may be nervous, e.g., going up and down stairs or going to an outside latrine.

At meal times, people with low vision can sit near the window or doorway so they can see what they are eating and when they have finished.

**Lines**. Many people with low vision find it hard to follow a row of text: they may not be able to scan the words easily, they may find it hard to know when they have got to the end of a row of text, or they may struggle to find the beginning of the next line. Partly blanking out the lines above and below the line being read, for example, using a reading slit (see page 10), makes the visible line of print easier to read. A reading slit can be made of black card with a rectangle cut out of it.

Lines can help with mobility and safety. For example, paint the edge of stairs in a contrasting colour, or put white paint on the top of stones which mark the path to a neighbour's home.

**Figure 4.**
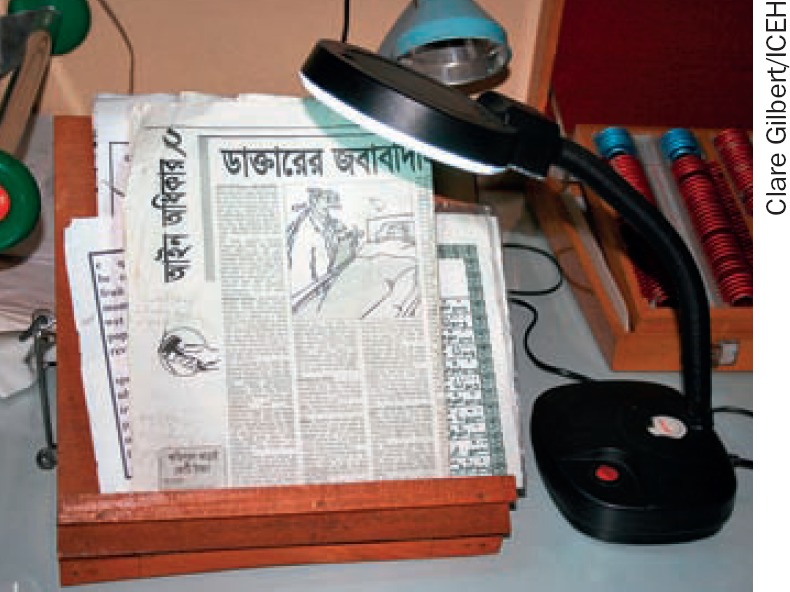
Reading stand with angled lamp

**Lift**. Figure 4 shows a locally made, foldable reading stand, lifts the page closer to the eyes and makes reading less tiring, particularly if magnifiers are used.

